# The Spinal Cord Injury – Quality of Life (SCI-QOL) measurement system: Development, psychometrics, and item bank calibration

**DOI:** 10.1179/2045772315Y.0000000035

**Published:** 2015-05

**Authors:** David S. Tulsky, Pamela A. Kisala

**Affiliations:** 1 Department of Physical Therapy, University of Delaware, College of Health Sciences, Newark, DE, USA; 2 Kessler Foundation Research Center, West Orange, NJ, USA

Though spinal cord injury (SCI) was historically regarded as an ailment not to be treated,^1^ medical, pharmaceutical, and technological advances in the 20th and 21st centuries have improved acute and long-term SCI rehabilitation outcomes. Consequently, SCI has become an increasingly common cause of long-term disability, with over 250 000 Americans^2,3^ and over 85 000 Canadians^4^ living with SCI. Traumatic SCI is a catastrophic injury that changes the lives of individuals in a split second. SCI is characterized by a broad and unique set of functional limitations and secondary complications that affect physical (e.g. altered urinary and bowel function,^5–7^ pressure ulcers,^8,9^ chronic and neuropathic pain) cognitive,^10,11^ emotional (e.g. depression,^12,13^ anxiety disorders),^14^ and social (e.g. unemployment)^15^ areas of health and functioning. Individuals who sustain SCI must adjust immediately to a new way of life that is often characterized by significant physical limitations, alterations to basic physiological functions, intense emotions, disruption of social relationships, and barriers to participating in their usual activities – essentially, every possible area of health-related quality of life (HRQOL). Individuals with SCI have described the secondary complications of SCI to be equally or even more troublesome than the primary functional limitations of SCI, such as the inability to walk.^16^ Furthermore, SCI is heterogeneous because the associated functional impairments and secondary medical issues are directly related to the location and neurological completeness of injury. An individual who sustains an American Spinal Injury Association (ASIA) Impairment Scale (AIS) grade D injury may be able to walk unassisted, while an individual with high-level and complete (AIS grade A) tetraplegia will be unable to move below the neck and will require constant mechanical ventilation. Due to the suddenness and severity of SCI, the wide range of potential secondary complications, and the diversity of functioning and complications within the population of individuals with SCI, healthcare professionals must assess a wide variety of areas of functioning, examine changes over time, and identify and mitigate potential risk factors. To do so, the healthcare provider must be able to measure and monitor a wide variety of issues that a person with SCI might experience. Until now, there have not been the proper tools to do so.

The lack of available SCI-relevant measurement instruments to conduct standardized, effective assessment of a wide variety of HRQOL domains has been rather disheartening. In 2001, Tulsky^17^ chaired a state-of-the-science conference for rehabilitation professionals that focused on the current state of quality of life measurement for individuals with disabilities. Several keynote addresses,^18,19^ as well as Tulsky and Rosenthal's synthesis^17^ of the conference, pointed out that, by and large, when HRQOL variables were utilized in clinical trials, rehabilitation researchers, including specialists in SCI medicine, were forced to use existing general scales that were developed and intended for the general population. These measurement tools did not capture areas of functioning that were important to individuals with physical disabilities, and often contained items that were irrelevant, inappropriate, or even offensive.^20–22^ Unfortunately, despite the flaws in these measurement tools for use with persons with disabilities, there was simply no alternative at that time. Outcomes measurement, in general, had not received the same level of attention and methodological rigor in rehabilitation as it had received in other fields of medicine.

In their summary of the conference talks, Tulsky and Rosenthal^23^ outlined a vision to improve rehabilitation outcomes measurement. This vision included John Ware's discussion of state-of-the-art assessment models using item response theory (IRT) that could provide flexible, dynamic and practical assessments,^24^ along with a paper providing detailed illustrations on how test items could be developed as ‘item banks’ for use across different groups of persons with disabilities.^25^ Though the vision was there, it was unclear if there would be support to implement such a strategy for individuals with SCI and other disabilities.

At the same time, rapid advances were taking place in the healthcare field in general. The first decade and a half of the new millennium was quickly establishing itself as a golden age for measurement, as new measurement methods and initiatives for healthcare and clinical trials research were being introduced. State-of-the-art measurement strategies from other fields such as education were being implemented in health research settings. In 2004, the National Institutes of Health (NIH) Common Fund (then called the NIH Roadmap) established the Patient Reported Outcomes Measurement Information System® (PROMIS®)^26^ and the National Institute of Neurological Disorders and Stroke (NINDS) embarked on a critical path to develop the Quality of Life in Neurological Disorders Measurement System (Neuro-QOL).^27^ The overarching goal was to develop state-of-the-art measurement scales to be used (for PROMIS®) across medical populations and (for Neuro-QOL) across individuals with Neurological Disorders. Unfortunately, these new initiatives did not target individuals with SCI.

Given the unique constellation of SCI-related symptoms, secondary complications, and potential alterations to social and emotional functioning, Dr. Tulsky and colleagues secured 2 grants to embark on a research initiative to fill this measurement gap. Dr. Tulsky received funding (in the form of an R-01) from the Eunice Kennedy Shriver National Institute on Child Health and Human Development/National Center on Medical Rehabilitation Research and the NINDS, as well a separate line of funding (in the form of a Model Systems Collaborative ‘Modular’ project) from the National Institute on Disability and Rehabilitation Research's Spinal Cord Injury Model System (SCIMS) program. An extensive network of collaboration between SCIMS, PROMIS, and Neuro-QOL investigators ensued and the research group set out to develop a psychometrically advanced measurement system that would be tailored for individuals with SCI and appropriate for use in both research and clinical settings. Ten years after the initial conference that Dr. Tulsky chaired, Tulsky, Carlozzi, and Cella^28^ painted a very different picture of the state of rehabilitation outcomes measurement. In contrast to the ‘doom and gloom’ picture from a decade earlier, Tulsky and colleagues reported that emerging, state-of-the-art measurement strategies were having a significant impact on the field.^26,27,29,30^ Tulsky and colleagues reported that new measurement initiatives, designed *specifically* for individuals with SCI (as well as for individuals with other chronic disabilities, such as traumatic brain injury) would provide SCI researchers and clinicians with valid and reliable outcome measures that addressed subjectively important issues to individuals with SCI.^16,31–34^ No longer was outcomes measurement an afterthought in SCI medicine. Instead, SCI outcomes researchers were leading the initiatives, including the Spinal Cord Injury – Quality of Life (SCI-QOL), which would transform outcomes measurement for rehabilitation research and practice.

The series of manuscripts that make up this special issue represent the culmination of the initial development of these SCI-specific item banks. These articles describe the detailed development work and psychometric calibration of the majority of SCI-QOL item banks. The purpose of this special issue is both to formally introduce the SCI-QOL to the field, and also to serve as a technical manual for use of the SCI-QOL item banks. The first manuscript in the issue provides a conceptual overview of the goals and outputs of the SCI-QOL project. Following the introductory overview,^35^ Tulsky, Kisala, Victorson, Choi, Gershon, Heinemann, and Cella^36^ provide a thorough description of the development and calibration methodology used across all SCI-QOL banks. They provide a description of five sequential phases of the SCI-QOL development work, each with unique goals, samples, and methods. Their paper provides detail on the research methodology that was used for the majority of manuscripts that follow in the special issue.

Following these introductory manuscripts, two manuscripts introduce SCI-QOL measures of secondary medical complications of SCI. First, Tulsky, Kisala, Tate, Spungen, and Kirshblum^37^ present the development of item banks to measure bowel management difficulties and bladder management difficulties, and a short scale to measure bladder complications. Next, Kisala, Tulsky, Choi, and Kirshblum present an IRT-calibrated scale to measure the impact of pressure ulcers on quality of life.

The next set of 8 manuscripts present the development of the SCI-QOL item banks related to emotional health following SCI. Kisala, Tulsky, Kalpakjian, Heinemann, Pohlig, Carle, and Choi^38^ present the SCI-QOL Anxiety item bank, which is a version of the PROMIS scale that has been tailored and optimized for the SCI population. The authors linked the SCI-QOL Anxiety scale with a frequently used brief measurement scale to assess anxiety in the general population (the Generalized Anxiety Disorder scale – 7 item version^39^; GAD-7). Post-traumatic stress, or psychological panic reactions due to traumatic events, have traditionally been classified as anxiety disorders. However, they have recently been reclassified as Trauma and Stress-or-Related Disorders under the new psychiatric/psychological classification system^40^ to reflect psychological reactions to a trigger that result from external traumatic events, such as exposure to actual or threatened death or serious injuries. An article by Kisala, Victorson, Pace, Heinemann, Choi, and Tulsky^41^ presents the SCI-QOL Trauma scale and will mark the first time that a scale has been developed to track this emotional reaction in individuals with SCI. The next two manuscripts are related to depressed feelings. Tulsky, Kisala, Kalpakjian, Bombardier, Pohlig, Heinemann, Carle, and Choi^42^ present the SCI-QOL Depression bank, which is an optimized version of the PROMIS scale for the SCI population. Given the common use of the Patient Health Questionnaire – 9 (PHQ-9)^43,44^ in individuals with SCI, the authors also report on the use of item response theory methods to convert scores on the PHQ-9 to SCI-QOL Depression scores. Qualitative input from individuals with SCI early in SCI-QOL development process led Tulsky and colleagues to develop a bank of items to assess the emotional components of a grief reaction, stemming from a sudden loss of functioning and difficulty adjusting to life, which differs from the traditional construct of depression. Kalpakjian, Tulsky, Kisala, and Bombardier^45^ present a new item bank to measure grief and loss after SCI. Next, two articles focus on positive psychological variables and emotional states. Bertisch, Kalpakjian, Kisala, and Tulsky^46^ present the SCI-QOL Positive Affect and Well-being item bank. This is a version of the Neuro-QOL Positive Affect and Well-being bank that has been optimized specifically for individuals with SCI and will provide researchers and clinicians with an efficient way to integrate constructs of positive affect and emotional well-being into SCI research and clinical practice. During the qualitative stage of the SCI-QOL's development, individuals with SCI described the necessity of accepting their injury and moving on with their ‘new’ life—of not just turning over a new page in life, but starting an entirely new book.^16^ The feedback underscored the importance of resilience following a traumatic injury such as SCI, and Victorson, Tulsky, Kisala, Kalpakjian, Weiland, and Choi^47^ present the development of the SCI-QOL Resilience item bank. The final two item banks focus on the feelings of appraisal, judgement, or stigmatization that individuals with SCI experience. Self-esteem refers to the cognitive, emotional, and evaluative perceptions of the self. Following SCI, individuals might experience self-generated negative emotions about themselves secondary to their injury. Kalpakjian, Tate, Kisala, and Tulsky^48^ present the SCI-QOL Self-esteem item bank. Parallel to such internal appraisals and due to the visible nature of a physical disability like SCI, there can be a stigmatizing effect of injury, especially in social settings. To measure these effects in individuals with SCI, Kisala, Tulsky, Pace, Victorson, Choi, and Heinemann^49^ describe the development and calibration of the SCI-QOL Stigma item bank. Several items were derived from the Neuro-QOL Stigma item bank and the SCI-QOL bank was placed on the Neuro-QOL metric.

The final manuscripts report on the SCI-QOL measures of social and physical functioning. Heinemann, Kisala, Hahn, and Tulsky^50^ report on the SCI-QOL Ability to Participate in Social Roles and Activities and the SCI-QOL Satisfaction with Social Roles and Activities item banks. Both of these item banks have utilized the items from the Neuro-QOL but have optimized the item banks for individuals with SCI. The final article, by Jette, Slavin, Ni, Kisala, Tulsky, Heinemann, Charlifue, Fyffe, Tate, Morse, Marino, Smith, and Williams.^51^ presents new enhancements to the SCI-QOL physical functioning scales (i.e. Spinal Cord Injury – Functional Index; SCI-FI).^31,52^ These item banks were developed using a separate (though parallel) sample and methodology and focus on how individuals perform activities with the use of assistive technology (AT). In contrast to the original SCI-FI items which ask participants about performing activities ‘without any devices or assistance,’ this paper by Jette *et al*. describes the development of the SCI-FI/AT – an enhancement of the original SCI-FI item banks to reflect the use of assistive technology when performing physical functions.

Each manuscript in this special issue presents one or more SCI-QOL item banks and reviews the included construct(s), item development/selection and reduction, item response theory analyses and further item reduction, calibration data, and brief, fixed-length ‘short form’ versions that have been developed for each bank. The SCI-QOL item banks provide a much richer assessment of functioning than traditional assessments and have included direct input from individuals with SCI throughout the development process. In each manuscript, we have presented all of the technical information related to the presented bank including the IRT calibration parameters (slope and threshold values), which can be useful if others want to develop a customized short forms or program a stand-alone computer adaptive test for a given item bank.

This series of manuscripts describes much of the SCI-QOL development work. However, as indicated by Tulsky, Kisala, Victorson, Choi, Gershon, Heinemann, and Cella,^36^ there are a few item pools/banks that have not been included in this special issue. Most notably, a detailed description of the SCI-QOL Pain Interference item bank and Pain Behavior scale, and the related calibration data, are not included here. Similarly, the development and calibration of the original 5 SCI-FI physical functioning item banks (i.e. Basic Mobility, Fine Motor function, Self-Care, Wheelchair Mobility, and Ambulation) have already been reported^31,52^ and as such are not included here. A SCI-QOL Independence item bank has been developed and is available on Assessment Center, though there is additional scaling work that has yet to be performed and it has not been included in this special issue. Finally, there are other pools of items that have been tested but not calibrated (e.g. Respiratory and Sexual Functioning subdomains). Due to the distribution of responses to these items, there was insufficient data to analyze these items with graded response model IRT analyses. Therefore, the Respiratory and Sexual Functioning item banks have not been calibrated and their description is beyond the scope of this special issue.

This special issue represents the contributions of many individuals. We extend our profound appreciation to the following individuals for their participation on this ambitious study.

First and foremost, we thank Dr. David Cella and Dr. David Victorson from the Northwestern University Department of Medical Social Sciences (MSS) for close partnership on this project from its initial stages through the entire item development and calibration process. Drs. Cella and Victorson transferred knowledge from the PROMIS and Neuro-QOL initiatives and ensured that we were conducting state-of-the-art measurement work in every aspect of the project. Dr. Victorson moderated all focus groups and could be counted on to assist us at every step along the way. Dr. Seung Choi and his team of psychometricians, Natalie McKinney and Tracy Podrabsky, conducted all of the item response theory analyses using the most meticulous procedures (and creating new procedures when there was no guide). Dr. Richard Gershon led the efforts to program the SCI-QOL item banks into Assessment Center assisted by several collaborators, including Dr. Nan Rothrock, Michael Bass, Maria Varela Diaz, Manpreet Lakhan, Monica Prudencio, and the entire MSS information technology team. Excellent technical support for our team's use of Assessment Center was provided by Warren Francis and Odessa Castro. Vitali Utsinovich provided materials and helped us understand the Neuro-QOL item banks and procedures. Jin-Shei Lai provided additional psychometric help and expertise. Dr. Cella, himself, provided inspiration for this project and provided the resources for the MSS scientists and staff and we remain eternally grateful.

Equally important were the contributions from all of our co-investigators from the SCI Model Systems for their hard work and significant contributions throughout the research project. We heartily thank Dr. Steven Kirshblum from the Kessler Institute of Rehabilitation and Dr. Trevor Dyson-Hudson, Dr. Denise Fyffe, and Dr. Amanda O'Brien Milleisen from the Kessler Foundation Research Center; Dr. Denise Tate, Dr. Claire Kalpakjian, and Martin Forchheimer from the University of Michigan; Dr. Susie Charlifue from Craig Hospital; Dr. Allen Heinemann from the Rehabilitation Institute of Chicago; Dr. Charles Bombardier and Dr. Dagmar Amtmann from the University of Washington; Dr. Alan Jette, Dr. Mary Slavin, and Dr. Pensheng Ni from Boston University; and Dr. Marcel Dijkers and Dr. Jeanne Zanca from Mount Sinai School of Medicine. We could not have dreamed of a better group of co-investigators with whom to have partnered over the last 9 years.

For a project of this magnitude, each institution brought their own army of data collectors and staff working effortlessly day after day on this project. At Kessler Foundation, Donna Servidio managed administrative aspects of the study and LeighAnn Martinez identified potential participants and assisted with all recruitment efforts. Data collectors included Rachel Byrne, Julia Stoumbos, Caitlin Seifert Miranda, Joseph McCabe, Erinn Stivala Nakahara, Michael Pino, Arielle Teitcher, Adrianna Maldonado, Brianne Smith, Heather McGowan, Katherine Czado Aquino, and Lila Inglima-Pereira; at RIC, the data collectors included Sara Jerousek, Ana Miskovic, Dustin Williams, Kyle Seanor, Brian Weiland, and Martha Bailey; at University of Michigan, Rachel Tocco helped with Assessment Center and data collectors included Jane Walters, Kate Donnelly, Siera Goodnight, Rachel Hartwig, Angela Miciura, Laury Elwell, Sam Leaf, and Sonya Sutherland; at Craig Hospital, data collectors included Amy Dannels-McClure, Susan Solnick, and Caroline Rose; and at University of Washington, Rana Salem helped with project coordination and data collectors included Kara Bogusz, Thayer Wild, Missy Takahashi, Matt Smith, and Meighan Rasley. At Boston University, data collectors included Diana Pernigotti and Vanessa Oliveira, and at Mt. Sinai, the data collection team included Marilyn Gomez, Jeannie Chan, Mila Babaev, Michelle Dziedzic, and Rana Searfoss. To develop the customized computer software that was utilized in the SCI-QOL calibration study, we thank Kunal Jain from VisionStream LLC.

We are very appreciative of the backing of the Kessler Foundation especially when this project was being developed. We thank Rodger DeRose, Dr. Mitchell Rosenthal, and Dr. Joel DeLisa, all of whom ensured that we have the appropriate financial and personnel support in the early phases of the project that established a strong foundation from which we could successfully complete a project of this magnitude. Matt Weiner and John Giraud provided incredible support from the business office to manage the project through its early years. Similarly, we thank our colleagues from the James J. Peters Department of Veterans Affairs Medical Center in the Bronx, NY: Dr. Ann Spungen, Kel Morin, and Shevana Swaby, for ensuring that the input of veterans was included so that their needs would be accounted for in the measurement system. Dr. Spungen is leading a VA sponsored study to validate SCI-QOL in a VA population.

Several investigators and staff worked on the early measurement initiatives that prepared us for future SCI-QOL funding and are deserving of our gratitude. Dr. Carol Miklos conducted our initial pilot interviews with individuals with SCI and Dr. Tamara Mills and Dr. Sandra Mercedes prepared initial item banks as we began to learn what issues were most important to individuals with SCI. Rachel Gold Tadduni, Kate Francis Hardy, and Amy Bullman Giles helped test these items in new pilot studies. This work set the foundation for SCI-QOL and helped us ‘hit the ground running’ once funding was secured. We would also like to thank the members of the Northern New Jersey SCI System Community Advisory Board who provided invaluable input throughout the first several years of the project.

Dr. Rita Bode, Dr. Adam Carle, and Dr. Ryan Pohlig provided psychometric and statistical help in the later stages of the project. Others who helped at various stages include Jerry Wright who helped with participant tracking, Dr. Jerry Slotkin who helped coordinate writing of the articles when the special issue was just being proposed, Brad Trumpower and Shale Maulana who helped prepare many of the tables included in this issue, Emily Buchanan who helped finalize the first set of manuscripts for submission, and Dr. Barton Palmer for conducting a preliminary peer review of some of the manuscripts. Similarly, we are grateful to Dr. Matthew Cohen for diligently reading all manuscripts and providing us with invaluable editorial comments. At the Journal of Spinal Cord Medicine, we would like to thank Dr. J. Scott Richards for serving as a guest editor for this issue and Stephen Cavanaugh for the hands-on management and editing of the issue. Finally, and most importantly, we would like to thank the approximately 1500 individuals with SCI who participated in the development, calibration, and reliability assessment phases of the study. The primary goal of the SCI-QOL was to ensure conceptual relevance by including input from individuals with SCI at every stage of the project. We will be forever indebted to the study participants who gave of their time and themselves by sharing their personal accounts of living with SCI, giving the research team a truly honest opinion of all of the preliminary items, and responding to seemingly endless sets of interview questions about some of their most personal feelings and experiences.

## References

[1] Hughes JT The Edwin Smith Surgical Papyrus: an analysis of the first case reports of spinal cord injuries. Paraplegia 1988;26(2):71–82. 3045730 10.1038/sc.1988.15

[2] Center NSCIS. Spinal Cord Injury Model Systems: 2013 Annual Report, Complete Public Version. Birmingham, Alabama; 2013.

[3] Christopher and Dana Reeve Foundation. One degree of separation: Paralysis and spinal cord injury in the United States. Short Hills, NJ: Christopher and Dana Reeve Foundation; 2009.

[4] Noonan VK, Fingas M, Farry A, Baxter D, Singh A, Fehlings MG, *et al.* Incidence and prevalence of spinal cord injury in Canada: a national perspective. Neuroepidemiology 2012;38(4):219–26. 22555590 10.1159/000336014

[5] Costa P, Perrouin-Verbe B, Colvez A Quality of life in spinal cord injury patients with urinary difficulties: Development and validation of qualiveen. Eur Urol 2001;39(1):107–13. 11173948 10.1159/000052421

[6] Fajardo NR, Pasiliao RV, Modeste-Duncan R, Creasey G, Bauman WA, Korsten MA Decreased colonic motility in persons with chronic spinal cord injury. Am J Gastroenterol 2003;98(1):128–34. 12526948 10.1111/j.1572-0241.2003.07157.x

[7] Hicken BL, Putzke JD, Richards JS Bladder management and quality of life after spinal cord injury. Am J Phys Med Rehabil 2001;80(12):916–22. 11821674 10.1097/00002060-200112000-00008

[8] Sugarman B Infection and pressure sores. Arch Phys Med Rehabil 1985;66(3):177–9. 3977573

[9] Richards J, Waites K, Chen Y, Kogos K, Schumitt M The epidemiology of secondary conditions following spinal cord injury. Top Spinal Cord Inj Rehabil 2004;10:15–29.

[10] Davidoff GN, Roth EJ, Richards JS Cognitive deficits in spinal cord injury: epidemiology and outcome. Arch Phys Med Rehabil 1992;73(3):275–84. 1543433

[11] Roth E, Davidoff G, Thomas P, Doljanac R, Dijkers M, Berent S, *et al.* A controlled study of neuropsychological deficits in acute spinal cord injury patients. Paraplegia 1989;27(6):480–9. 2608301 10.1038/sc.1989.75

[12] Hancock KM, Craig AR, Dickson HG, Chang E, Martin J Anxiety and depression over the first year of spinal cord injury: a longitudinal study. Paraplegia 1993;31(6):349–57. 8336997 10.1038/sc.1993.59

[13] Fuhrer JM, Rintala DH, Hart KA Depressive symptomatology in persons with spinal cord injury who reside in the community. Arch Phys Med Rehabil 1993;74:255–60. 8439251

[14] Kennedy P, Rogers BA Anxiety and depression after spinal cord injury: A longitudinal analysis. Arch Phys Med Rehabil 2000;81(7):932–7. 10896007 10.1053/apmr.2000.5580

[15] Tomassen P, Post M, van Asbeck F Return to work after spinal cord injury. Spinal Cord 2000;38:51–5. 10762198 10.1038/sj.sc.3100948

[16] Tulsky DS, Kisala PA, Victorson D, Tate D, Heinemann AW, Amtmann D, *et al.* Developing a contemporary patient-reported outcomes measure for spinal cord injury. Arch Phys Med Rehabil 2011;92(10 Suppl):S44–51. 21958922 10.1016/j.apmr.2011.04.024 PMC6309317

[17] Tulsky DS, Rosenthal M Quality of life measurement in rehabilitation medicine: building an agenda for the future. Arch Phys Med Rehabil 2002;83(12 Suppl 2):S1–3. 12474165 10.1053/apmr.2002.36954

[18] Tate D, Kalpakjian C, Forchheimer M Quality of life issues in individuals with spinal cord injury. Arch Phys Med Rehabil 2002;83(Suppl. 2):S1–8. 12474168 10.1053/apmr.2002.36835

[19] Hays RD, Hahn H, Marshall G Use of the SF-36 and other health-related quality of life measures to assess persons with disabilities. Arch Phys Med Rehabil 2002;83(12 Suppl 2):S4–9. 12474166 10.1053/apmr.2002.36837

[20] Andresen EM, Meyers AR Health-related quality of life outcomes measures. Arch Phys Med Rehabil 2000;81(12 Suppl 2):S30–45. 11128902 10.1053/apmr.2000.20621

[21] Meyers AR, Andresen EM, Hagglund KJ A model of outcomes research: spinal cord injury. Arch Phys Med Rehabil 2000;81(12 Suppl 2):S81–90. 11128907 10.1053/apmr.2000.20629

[22] Andresen EM, Fouts BS, Romeis JC, Brownson CA Performance of health-related quality-of-life instruments in a spinal cord injured population. Arch Phys Med Rehabil 1999;80(8):877–84. 10453762 10.1016/s0003-9993(99)90077-1

[23] Tulsky DS, Rosenthal M Measurement of quality of life in rehabilitation medicine: emerging issues. Arch Phys Med Rehabil 2003;84(4 Suppl 2):S1–2. 12692765 10.1053/apmr.2003.50203

[24] Ware JE Conceptualization and measurement of health-related quality of life: comments on an evolving field. Arch Phys Med Rehabil 2003;84(4):S43–S51. 12692771 10.1053/apmr.2003.50246

[25] Bode RK, Lai JS, Cella D, Heinemann AW Issues in the development of an item bank. Arch Phys Med Rehabil 2003;84(4):S52–S60. 12692772 10.1053/apmr.2003.50247

[26] Cella D, Riley W, Stone A, Rothrock N, Reeve B, Yount S, *et al.* The Patient-Reported Outcomes Measurement Information System (PROMIS) developed and tested its first wave of adult self-reported health outcome item banks: 2005–2008. J Clin Epidemiol 2010;63(11):1179–94. 20685078 10.1016/j.jclinepi.2010.04.011 PMC2965562

[27] Cella D, Nowinski C, Peterman A, Victorson D, Miller D, Lai J-S, *et al.* The neurology quality-of-life measurement initiative. Arch Phys Med Rehabil 2011;92(10, Supplement):S28–S36. 21958920 10.1016/j.apmr.2011.01.025 PMC3193028

[28] Tulsky DS, Carlozzi NE, Cella D Advances in outcomes measurement in rehabilitation medicine: current initiatives from the National Institutes of Health and the National Institute on Disability and Rehabilitation Research. Arch Phys Med Rehabil 2011;92(10 Suppl 1):S1–6. 21958917 10.1016/j.apmr.2011.07.202 PMC4425123

[29] Quatrano LA, Cruz TH Future of outcomes measurement: impact on research in medical rehabilitation and neurologic populations. Arch Phys Med Rehabil 2011;92(10 Suppl):S7–11. 21958925 10.1016/j.apmr.2010.08.032

[30] Gershon RC, Wagster MV, Hendrie HC, Fox NA, Cook KF, Nowinski CJ NIH toolbox for assessment of neurological and behavioral function. Neurology 2013;80(Suppl 3):S2–S6. 23479538 10.1212/WNL.0b013e3182872e5f PMC3662335

[31] Tulsky DS, Jette AM, Kisala PA, Kalpakjian C, Dijkers MP, Whiteneck G, *et al.* Spinal cord injury-functional index: item banks to measure physical functioning in individuals with spinal cord injury. Arch Phys Med Rehabil 2012;93(10):1722–32. 22609299 10.1016/j.apmr.2012.05.007 PMC3910090

[32] Carlozzi NE, Tulsky DS, Kisala PA Traumatic brain injury patient-reported outcome measure: identification of health-related quality-of-life issues relevant to individuals with traumatic brain injury. Arch Phys Med Rehabil 2011;92(10 Suppl):S52–60. 21958923 10.1016/j.apmr.2010.12.046

[33] Slavin MD, Kisala PA, Jette AM, Tulsky DS Developing a contemporary functional outcome measure for spinal cord injury research. Spinal Cord 2010;48(3):262–7. 19841635 10.1038/sc.2009.131

[34] Amtmann D, Cook KF, Johnson KL, Cella D The PROMIS initiative: involvement of rehabilitation stakeholders in development and examples of applications in rehabilitation research. Arch Phys Med Rehabil 2011;92(10):S12–S9. 21958918 10.1016/j.apmr.2011.04.025 PMC3668854

[35] Tulsky DS, Kisala PA, Victorson D, Tate DG, Heinemann AW, Charlifue S, *et al.* Overview of the Spinal Cord Injury - Quality of Life (SCI-QOL) measurement system. J Spinal Cord Med 2015;38(3):257–69. 26010962 10.1179/2045772315Y.0000000023 PMC4445018

[36] Tulsky DS, Kisala PA, Victorson D, Choi SW, Gershon R, Heinemann AW, *et al.* Methodology for the development and calibration of the SCI-QOL item banks. J Spinal Cord Med 2015;38(3):270–87. 26010963 10.1179/2045772315Y.0000000034 PMC4445019

[37] Tulsky DS, Kisala PA, Tate DG, Spungen AM, Kirshblum SC Development and psychometric characteristics of the SCI-QOL Bladder Management Difficulties and Bowel Management Difficulties item banks and short forms and the SCI-QOL Bladder Complications scale. J Spinal Cord Med 2015;38(3):288–302. 26010964 10.1179/2045772315Y.0000000030 PMC4445020

[38] Kisala PA, Tulsky DS, Kalpakjian CZ, Heinemann AW, Pohlig RT, Carle A, *et al.* Measuring anxiety after spinal cord injury: Development and psychometric characteristics of the SCI-QOL Anxiety item bank and linkage with GAD-7. J Spinal Cord Med 2015;38(3):315–25. 26010966 10.1179/2045772315Y.0000000029 PMC4445022

[39] Spitzer RL, Kroenke K, Williams JB, Lowe B A brief measure for assessing generalized anxiety disorder: the GAD-7. Arch Intern Med 2006;166(10):1092–7. 16717171 10.1001/archinte.166.10.1092

[40] Association AP Diagnostic and statistical manual of mental disorders. 5th ed Washington, DC: American Psychiatric Association; 2013.

[41] Kisala PA, Victorson D, Pace N, Heinemann AW, Choi SW, Tulsky DS Measuring psychological trauma after spinal cord injury: Development and psychometric characteristics of the SCI-QOL Psychological Trauma item bank and short form. J Spinal Cord Med 2015;38(3):326–34. 26010967 10.1179/2045772315Y.0000000022 PMC4445023

[42] Tulsky DS, Kisala PA, Kalpakjian CZ, Bombardier CH, Pohlig RT, Heinemann AW, *et al.* Measuring depression after spinal cord injury: Development and psychometric characteristics of the SCI-QOL Depression item bank and linkage with PHQ-9. J Spinal Cord Med 2015;38(3):335–46. 26010968 10.1179/2045772315Y.0000000020 PMC4445024

[43] Kroenke K, Spitzer RL, Williams JB The PHQ-9: validity of a brief depression severity measure. J Gen Intern Med 2001;16(9):606–13. 11556941 10.1046/j.1525-1497.2001.016009606.x PMC1495268

[44] Williams RT, Heinemann AW, Bode RK, Wilson CS, Fann JR, Tate DG Improving measurement properties of the patient health questionnaire-9 with rating scale analysis. Rehabil Psychol 2009;54(2):198–203. 19469610 10.1037/a0015529

[45] Kalpakjian CZ, Tulsky DS, Kisala PA, Bombardier CH Measuring grief and loss after spinal cord injury: Development, validation and psychometric characteristics of the SCI-QOL Grief and Loss item bank and short form. J Spinal Cord Med 2015;38(3):347–55. 26010969 10.1179/2045772315Y.0000000015 PMC4445025

[46] Bertisch H, Kalpakjian CZ, Kisala PA, Tulsky DS Measuring positive affect and well-being after spinal cord injury: Development and psychometric characteristics of the SCI-QOL Positive Affect and Well-being item bank and short form. J Spinal Cord Med 2015;38(3):356–65. 26010970 10.1179/2045772315Y.0000000024 PMC4445026

[47] Victorson D, Tulsky DS, Kisala PA, Kalpakjian CZ, Weiland B, Choi SW Measuring resilience after spinal cord injury: Development, validation and psychmetric characteristics of the SCI-QOL Resilience item bank and short form. J Spinal Cord Med 2015;38(3):366–76. 26010971 10.1179/2045772315Y.0000000016 PMC4445027

[48] Kalpakjian CZ, Tate DG, Kisala PA, Tulsky DS Measuring self-esteem after spinal cord injury: Development, validation and psychometric characteristics of the SCI-QOL Self-esteem item bank and short form. J Spinal Cord Med 2015;38(3):377–85. 26010972 10.1179/2045772315Y.0000000014 PMC4445028

[49] Kisala PA, Tulsky DS, Pace N, Victorson D, Choi SW, Heinemann AW Measuring stigma after spinal cord injury: Development and psychometric characteristics of the SCI-QOL Stigma item bank and short form. J Spinal Cord Med 2015;38(3):386–96. 26010973 10.1179/1079026815Z.000000000410 PMC4445029

[50] Heinemann AW, Kisala PA, Hahn EA, Tulsky DS Development and psychometric characteristics of the SCI-QOL Ability to Participate and Satisfaction with social roles and activities item banks and short forms. J Spinal Cord Med 2015;38(3):397–408. 26010974 10.1179/2045772315Y.0000000028 PMC4445030

[51] Jette AM, Slavin MD, Ni P, Kisala PA, Tulsky DS, Heinemann AW, *et al.* Development and initial evaluation of the SCI-FI/AT. J Spinal Cord Med 2015;38(3):409–18. 26010975 10.1179/2045772315Y.0000000003 PMC4445031

[52] Jette AM, Tulsky DS, Ni P, Kisala PA, Slavin MD, Dijkers MP, *et al.* Development and initial evaluation of the spinal cord injury-functional index. Arch Phys Med Rehabil 2012;93(10):1733–50. 22609635 10.1016/j.apmr.2012.05.008

